# A Case of Neuroleptic Malignant Syndrome Presenting as Anxiety

**DOI:** 10.7759/cureus.35892

**Published:** 2023-03-08

**Authors:** Cameron G Hanson, Amit Chopra

**Affiliations:** 1 Department of Emergency Medicine, Henry Ford Health System, Macomb, USA

**Keywords:** common emergency department complaints, neuro-psychiatric, mood and anxiety, neuroleptic malignant syndrome (nms), emergency psychiatry

## Abstract

Neuroleptic malignant syndrome (NMS) is a rare but potentially lethal complication of dopamine antagonist use. A 34-year-old male presented to the emergency department with a chief complaint of feeling anxious for the past several days. He presented with his family who helped provide history as he had become less communicative over the preceding two days. It was revealed that the patient had a recent psychiatric hospitalization for suspected new-onset psychosis and was discharged six days prior to his presentation. It was reported that the patient was discharged with unknown psychiatric medications but stopped taking them two days prior because he felt they were increasing his anxiety. On physical examination, the patient was found to have upper extremity rigidity and appeared tremulous. A review of records revealed that the patient was discharged from inpatient psychiatric treatment on dual antipsychotic therapy. With this information, the patient met the diagnostic criteria for NMS. He was hospitalized and his symptoms resolved following treatment. Without the knowledge of antipsychotic use, the diagnosis of a serious, life-threatening condition may have been missed. Our case highlights an important but occasionally overlooked aspect of evaluating a patient in the emergency department, namely, outside chart and documentation reviewing.

## Introduction

Psychiatric complaints are frequently seen in emergency departments and are becoming increasingly common [1]. Anxiety and its related conditions are a common chief complaint and may affect up to 16% of the US population and have been associated with medical comorbidities [2]. Additionally, it has been demonstrated that anchoring and affect bias among emergency physicians may lead to an inadequate medical investigation, potentially leading to missed diagnoses [3]. Neuroleptic malignant syndrome (NMS) is a serious, potentially life-threatening emergency that needs to be identified early to initiate treatment. Most commonly, first-generation antipsychotics, including haloperidol, fluphenazine, and perphenazine, are associated with the development of this condition [4]. With the increase in the prevalence of second-generation antipsychotics, including olanzapine, it is important to consider that these medications can also lead to NMS [5]. In this case report, the authors detail a case of NMS which was initially obfuscated by subtle symptomatology and missing elements of the patient’s history. This case highlights the importance of chart reviewing and outside record investigation, an often overlooked aspect of emergency medicine.

## Case presentation

A 34-year-old male presented to the emergency department with a chief complaint of anxiety. The patient had presented with a family member who provided a majority of the history for the patient. Per the family member, for the past two days, the patient had been complaining of increased feelings of anxiousness and restlessness. Reportedly, the patient had a history of new-onset psychosis around one month prior. At this time, symptoms included similar feelings of restlessness, responding to internal stimuli, disorganized behavior, and paranoia. Following this new diagnosis, the patient was treated at an inpatient psychiatric facility and discharged around one week prior to his presentation. The family member stated that the patient had no other prior significant medical history including substance use. It was noted that following discharge from inpatient psychiatric treatment, he had significant improvement in his symptoms. The family did not know what medications he was discharged on but stated that he was not currently taking any medications. The patient confirmed he was taking “a few” medications after discharge but abruptly stopped two days after discharge. He had stated that he believed they were causing him worse anxiety and wanted to try to manage his psychiatric symptoms “holistically without medication.” He denied any auditory hallucinations or paranoia. Prior to his first episode of psychosis, the patient had no mental health concerns.

On examination, the patient’s heart rate was 108 beats per minute, temperature was 99.8°F, and he had a blood pressure of 165/95 mmHg. Physical examination revealed a well-appearing male who was mildly diaphoretic and tremulous. He was minimally communicative, however, repeatedly stated that he felt “anxious.” The patient’s arms were noted to be flexed and internally rotated with his hands clasped together with rigidity. His family had noted that he had been doing this intermittently over the past several days and stated that the patient said this helped him feel “less anxious.” Initial laboratory tests ordered included a complete blood count, complete metabolic panel, lactic acid, and magnesium. These tests were unremarkable apart from mildly elevated aspartate transaminase and alanine transaminase (Table 1).

**Table 1:** The patient’s initial laboratory findings. CO_2_ = carbon dioxide; BUN = blood urea nitrogen; AST = aspartate transaminase; ALT = alanine transaminase

Laboratory test	Patient’s value	Normal reference range
Sodium	138	136 to 144 mEq/L
Potassium	4.1	3.7 to 5.2 mEq/L
Chloride	104	96 to 106 mmol/L
CO_2_	27	23 to 29 mmol/L
Anion gap	7	4 to 12 mEq/L
BUN	9	6 to 20 mg/dL
Creatinine	1.01	0.6 to 1.3 mg/dL
Glucose	87	70 to 100 mg/dL
Calcium	9.1	8.5 to 10.2 mg/dL
AST	68	8 to 33 U/L
ALT	49	4 to 36 U/L
Total bilirubin	1.0	0.1 to 1.2 mg/dL

At this time, attention was turned to the patient’s past medical records and history pertaining to his recent psychiatric hospitalization. A review of his records revealed that the patient was seen at an outside emergency department for symptoms including nonsensical words and responding to internal stimuli around one month prior. Medical workup including computerized tomography of the head without contrast and laboratory work at that time was unremarkable. Psychiatry was consulted and recommended initiation of risperidone 1 mg nightly, along with inpatient psychiatric treatment. The patient was subsequently transferred to an inpatient psychiatric treatment facility. He was hospitalized over the next 28 days. Specific details on medication titrations were not available from hospitalization records. He was noted to make a significant improvement in mental status and functionality and, per the inpatient psychiatrist’s recommendations, was discharged on olanzapine 5 mg nightly, haloperidol 5 mg every 12 hours, and benztropine 0.5 mg every 12 hours for dystonia prophylaxis. Unfortunately, the rationale for this regimen was not provided in the hospitalization documentation. Per the discharge summary of the inpatient psychiatric facility, the patient had not displayed any behaviors concerning for extrapyramidal symptoms, dystonic reactions, or NMS while on this medication regimen for one week prior to discharge.

Six days following his discharge, the patient presented back to the emergency department with the above complaints. After obtaining further medication history, including recent history of dual antipsychotic use, a creatine phosphokinase (CPK) was ordered which was found to be elevated (Table 2).

**Table 2:** Patient’s additional laboratory results after a review of prior medication history. CPK = creatine phosphokinase

Laboratory test	Patient’s value	Normal reference range
CPK	2,566	10 to 120 µg/L

The patient was subsequently started on intravenous fluid rehydration and transferred to the intensive care unit where he made significant progress. Psychiatry evaluation was performed while hospitalized, and no new medications were recommended at that time as rigidity and feelings of anxiousness had resolved and the patient had not experienced any symptoms of psychosis. The patient was discharged from the hospital three days later and was referred to outpatient psychiatry evaluation.

## Discussion

The Diagnostic and Statistical Manual of Mental Disorders 5th Edition (DSM-5) diagnostic criteria of NMS include exposure to a dopamine antagonist along with major and minor symptoms (Table 3).

**Table 3:** The Diagnostic and Statistical Manual of Mental Disorders 5th Edition diagnostic criteria for NMS. CPK = creatine phosphokinase; NMS = neuroleptic malignant syndrome

Feature	Description
Major symptoms	Rigidity hyperthermia (>100.4°F), diaphoresis, exposure to dopamine antagonist within 72 hours prior to the development of symptoms
Minor symptoms	Autonomic nervous system: Tachycardia, hypertonia, excessive salivation, tachypnea, shortness of breath. Mental status: altered level of consciousness, delirium, stupor. Motor symptoms: tremor, akinesia, dystonia, trismus, myoclonia. Laboratory findings: leukocytosis, increased CPK, increased creatinine, hypoxia, increased liver function tests
Exclusion criteria	The above symptoms are not due to another substance or neurologic or medical condition

Our workup and the patient’s subsequent medical course did not find another etiology for his presenting symptoms. Based on the above criteria, the patient had evidence of tachycardia, altered level of consciousness per family (minimally communicative), dystonia, and increased CPK and liver function tests, making the diagnosis of NMS the most likely.

For any patient taking antipsychotics and neuroleptic medications, drug reaction should be on the differential of possible etiologies of worsening psychiatric complaints. These reactions can include acute dystonia, acute akathisia, tardive dyskinesia, serotonin syndrome, and NMS [4,6]. In our case, the patient displayed findings more indicative of NMS based on DSM-5 criteria. This syndrome can occur within hours of exposure to a neuroleptic agent; however, on average, the time of onset is between four and 14 days, with up to 90% occurring within the first 10 days of starting these medications [7]. Additionally, patients with this syndrome may not initially develop rigidity or hyperthermia, as seen in our case [8]; however, laboratory abnormalities may help with making the diagnosis [9]. Although the patient did meet some of the criteria of DSM-5, in the absence of known antipsychotic use, the diagnosis may have been missed. The above case highlights the importance of collateral information gathering and medical chart review.

## Conclusions

In this case, the authors highlight a few key factors in the evaluation of a patient with a psychiatric complaint in the emergency department. The patient’s chief complaint was most likely not related to a previously diagnosed psychiatric disorder, as symptoms resolved following treatment for NMS, but instead due to an underlying medical condition induced by his previous treatment. Although he had stopped taking his medication prior to his presentation, the patient was subsequently diagnosed with NMS due to the residual effects of his medications. It is important for emergency physicians to remember that not all psychiatric complaints are purely due to psychiatric etiologies, and reviewing every patient’s prior medication history from outside charts or documentation is an important aspect of evaluating a patient.

## References

[1] Madsen TE, Bennett A, Groke S (2009). Emergency department patients with psychiatric complaints return at higher rates than controls. West J Emerg Med.

[2] Aquin JP, El-Gabalawy R, Sala T, Sareen J (2017). Anxiety disorders and general medical conditions: current research and future directions. Focus (Am Psychiatr Publ).

[3] Park DB, Berkwitt AK, Tuuri RE, Russell WS (2014). The hateful physician: the role of affect bias in the care of the psychiatric patient in the ED. Am J Emerg Med.

[4] Strawn JR, Keck PE Jr, Caroff SN (2007). Neuroleptic malignant syndrome. Am J Psychiatry.

[5] Kogoj A, Velikonja I (2003). Olanzapine induced neuroleptic malignant syndrome--a case review. Hum Psychopharmacol.

[6] Werneke U, Jamshidi F, Taylor DM, Ott M (2016). Conundrums in neurology: diagnosing serotonin syndrome - a meta-analysis of cases. BMC Neurol.

[7] Tse L, Barr AM, Scarapicchia V, Vila-Rodriguez F (2015). Neuroleptic malignant syndrome: a review from a clinically oriented perspective. Curr Neuropharmacol.

[8] Carbone JR (2000). The neuroleptic malignant and serotonin syndromes. Emerg Med Clin North Am.

[9] Hermesh H, Manor I, Shiloh R, Aizenberg D, Benjamini Y, Munitz H, Weizman A (2002). High serum creatinine kinase level: possible risk factor for neuroleptic malignant syndrome. J Clin Psychopharmacol.

